# The Impact of Lunch Timing on Nap Quality

**DOI:** 10.3390/clockssleep6030027

**Published:** 2024-08-05

**Authors:** Jennifer E. Fudge, Emily T. Peterson, Shae-Lynn M. Koe, Hans C. Dringenberg

**Affiliations:** Department of Psychology, Queen’s University, Kingston, ON K7L 3N6, Canada; 17etp2@queensu.ca (E.T.P.); shaelynn.koe@queensu.ca (S.-L.M.K.)

**Keywords:** sleep hygiene, EEG analysis, food intake, circadian rhythm

## Abstract

Purpose: Previous research has established that food intake is a biological regulator of the human sleep–wake cycle. As such, the timing of eating relative to sleep may influence the quality of sleep, including daytime naps. Here, we examine whether the timing of lunch (1 h vs. 2 h interval between lunch and a napping opportunity) impacts the quality of an afternoon nap. Methods: Using a randomized within-subject design over two separate experimental sessions (7 days apart), participants (*n* = 40, mean age = 25.8 years) consumed lunch 1 h and 2 h prior to an afternoon nap opportunity. Polysomnography and subjective self-reports were used to assess sleep architecture, sleepiness levels, and nap quality. Results: Results revealed no significant differences in subjective ratings of sleep quality and sleepiness, or in sleep architecture (total sleep time, sleep efficiency, sleep onset latency, sleep stages) between the 1 h and 2-h lunch conditions. Conclusions: All sleep measures were similar when napping followed eating by either 1 h or 2 h, suggesting that eating closer to nap onset may not negatively impact sleep architecture and quality. Future research should continue to identify conditions that improve nap quality, given the well-documented benefits of naps to reduce sleep pressure and improve human performance.

## 1. Plain Language Summary

Eating prior to sleep may influence sleep quality. We found that sleep architecture, rating of nap quality, and levels of sleepiness were similar when participants consumed lunch either 1 h or 2 h prior to an afternoon nap. Thus, for regular nappers and within the parameters of the current study, the timing of a meal relative to the start of an afternoon nap may not have a major impact on sleep characteristics and subjective feelings of sleepiness.

## 2. Introduction

Sleep constitutes an important state of rest and recovery of the body, thus contributing to overall physical and mental health. Consequently, insufficient sleep (often considered to be less than 7 h/night) is associated with numerous adverse health outcomes, including cardiovascular disease, metabolic disturbances and obesity, mental health problems (e.g., depression), neurodegenerative diseases, reduced immune responses, as well as impairments in mood and cognitive functioning (e.g., attention, learning, and memory; see [1,2] for current reviews). 

Given the importance of adequate sleep, it is not surprising that researchers and healthcare professionals have attempted to identify factors that influence (positively or adversely) sleep quality; one of these factors is food intake [3,4]. It has long been recognized that the duration, quality, and timing of sleep can exert powerful effects on appetite regulation. Insufficient sleep and/or circadian misalignment (e.g., sleep at times that do not match a person’s circadian rhythm and chronotype) are associated with changes in the release of appetite-regulating hormones such as ghrelin and leptin, known to promote and suppress appetite, respectively [5,6]. Further, the relation of sleep and food intake is bidirectional, in that food intake itself is thought to affect sleep quality. For example, a number of studies have examined whether eating prior to sleep onset impacts the quality of overnight sleep. Some evidence, mostly based on self-reports of meal consumption and sleep parameters, suggests that eating prior to sleep can negatively impact sleep quality. Eating-related disturbances in several sleep parameters have been reported, including the following: reduced sleep duration [7,8], increased wake after sleep onset (WASO) [7,9], increased sleep onset latency (SOL) [10], altered distributions of slow wave sleep (SWS) and light sleep [11], as well as poorer self-reported sleep quality [12,13]. Similar effects of eating on subsequent sleep quality have also been noted in studies of Muslims during Ramadan, a period when all eating is restricted to the evening (after sunset). The available evidence indicates an increased occurrence of multiple sleep disruptions that appear to be related to the altered eating schedule during Ramadan, even though additional factors (e.g., larger meal sizes) may also contribute to these effects [14,15,16]. Based on the evidence summarized above, reputable health and sleep organizations now recommend that, to improve sleep hygiene and sleep quality, late (and heavy) evening meals should be avoided (e.g., “Don’t Dine Late”, Sleep Foundation [17]; “Don’t eat a large meal before bedtime”, American Academy of Sleep Medicine/Sleep Education [18]).

It is important to acknowledge that the effects of food intake on sleep are highly complex and influenced by a number of (likely interacting) variables, including the following: meal volume and temperature, caloric content, macro- and micro-nutrient content, as well as the timing of meal consumption relative to sleep. Some of these factors have been examined in greater detail (e.g., the role of caloric and nutrient content—for examples, see [10,19,20,21,22])—while others have received relatively little attention. Specifically, very few studies to date have investigated the specific role of meal timing relative to bedtime on sleep, particularly by means of experimental studies conducted under controlled laboratory conditions. Further, the available evidence on the effect of the time interval between eating and sleep on sleep quality is inconsistent, perhaps due to the use of retrospective self-reports and the wide variety of questionnaire instruments used in these studies. Using survey data collected in a representative sample of U.S. residents, Iao et al. [7] found that eating closer to bedtime was associated with longer WASO and sleep duration, the latter perhaps a compensatory mechanism to counter the increased WASO. Chung et al. [9] conducted an online survey and compared sleep measures between participants who consumed a meal within 3 h or more than 3 h of their bedtime. The results showed that eating within 3 h of sleeping was related to more frequent nocturnal awakenings. In one of the few laboratory-based studies, Afaghi et al. [10] found evidence for a reduced SOL for a meal consumed 3 h prior to bedtime relative to a 1 h interval.

Overall, the work on the timing of eating and sleep summarized above is consistent with the notion that food intake closer to bedtime exerts a negative impact on sleep. There is, however, evidence that does not support this general conclusion. Falkenberg et al. [20] showed, in elite athletes, each additional hour between the main evening meal (dinner) and the last evening meal (a later snack) and bedtime was associated with a reduction of total sleep time (TST) by 8 and 6 min, respectively. These results led the researchers to emphasize the importance of adequate food intake prior to sleep, rather than the potential disruptive effects of eating on subsequent sleep [20]. Recently, Duan et al. [11] examined whether sleep architecture differed between participants who ate a meal 1 or 5 h prior to bedtime. The results revealed that, relative to 5 h, participants in the 1 h condition showed a slight, but statistically significant increase in deep sleep (delta power) during the first half of the night, followed by an increased light sleep in the second half of the night. Duan et al. [11] conclude that, contrary to common assumptions, eating closer to bedtime may not have adverse effects on sleep architecture.

In summary, the available evidence regarding the impact of the time interval between eating and sleep on sleep architecture and quality is contradictory, and very few studies have investigated this topic under controlled laboratory conditions. Further, the work conducted so far has focused on the impact of food intake on overnight sleep, thus neglecting the question of whether meal timing can affect nap quality. This is an important avenue of research, since napping is a relatively common and recommended practice that affords important beneficial effects on health and cognitive performance, particularly for populations that suffer from poor overnight sleep (e.g., elderly, sleep disorder patients), or from sleep debt due to imposed sleep schedules and/or restrictions (e.g., shift workers, healthcare professionals) [23,24,25,26,27,28].

The current study aimed to fill the gaps in the literature by examining the impact of different time intervals between consuming lunch and a subsequent afternoon nap on sleep (nap) architecture, self-perceived nap quality, and self-perceived sleepiness before and after the nap. The main objective of the experiments reported below was to assess if increasing the time interval (from 1 h to 2 h) between consuming lunch and an afternoon nap alters objective (polysomnography) and subjective (self-report) measures of sleep quality in a sample of adults. In the longer term, we hope that the present work will facilitate the development of sleep hygiene guidelines and recommendations specifically for afternoon naps, given the benefits of napping for various populations, as discussed above.

## 3. Results

### 3.1. Participant Characteristics

A total of 40 participants, recruited from the Kingston, Ontario area, completed the study. The final sample consisted of 29 self-identified females (72.5%) and 11 self-identified males (27.5%) and had an average age of about 25.8 years (*M* = 25.8, *SD* = 10.2) (see Section 5 for details on inclusion and exclusion criteria). Across all participants, the baseline (pre-nap) level of sleepiness as assessed by the Stanford Sleepiness Scale (SSS) was *M* = 3.2 (*SD* = 1.2). In the demographic survey, the majority of participants (24, 60%) agreed with the statement “I generally sleep well, but occasionally suffer from lack of enough sleep”; 10 (25%) participants agreed with “My sleep is quite mixed; at times I sleep well, but at other times, I suffer from sleep loss and tiredness”; 5 (12.5%) participants agreed with “I have no trouble sleeping and usually feel that I get enough sleep and am well-rested”; and 1 participant (2.5%) agreed with “I am a poor sleeper and it is rare for me to feel well-rested”.

### 3.2. Sleep Architecture

**Figure 1:**
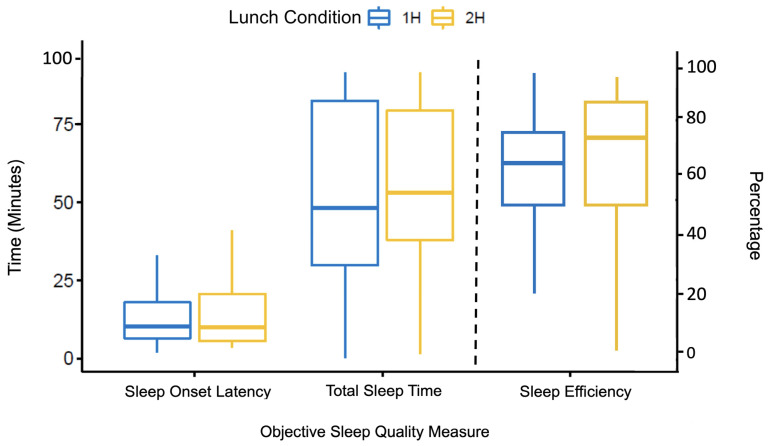
Objective sleep quality measures by condition. Sleep Onset Latency = the number of minutes participants took to fall asleep; Total Sleep Time = the total number of minutes the participant spent asleep; Sleep Efficiency = total sleep time divided by the total amount of time in minutes that the participant spent in bed (please note that bars to the left and right of the dotted line refer to the left and right y-axis, respectively).

To assess sleep architecture (Table 1), total sleep time (TST) was calculated by adding up the total number of minutes a participant was asleep, while sleep onset latency (SOL) was defined as the number of minutes from the time the experimenter had instructed the participants to start their nap, left the room, and turned off the lights to the onset of sleep, based on the polysomnographic record (scored off-line). Two participants were not able to fall asleep during the completion of their 1 h condition. For these participants, SOL was calculated as the number of minutes the participant spent in bed (the maximal nap opportunity allowed was 2 h). As an additional measure, sleep efficiency (SE) was calculated by dividing the total amount of time (in minutes) the participant was asleep by the total amount of time (in minutes) the participant was in bed. As displayed in Table 1 and Figure 1, the statistical analyses revealed that none of the sleep measures differed between the 1 h and 2 h conditions; SOL: *t*(39) = 0.092, *p* = 0.927; TST, *t*(39) = −1.442, *p* = 0.259; SE, *t*(39) = −1.368, *p* = 0.179.

**Figure 2:**
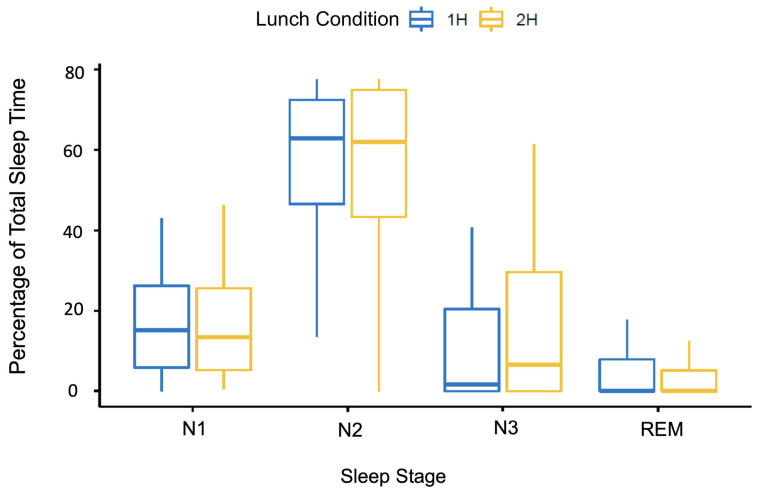
Percentage of total sleep time spent in each sleep stage. N1 = Non-REM 1; N2 = Non-REM 2; N3 = Non-REM 3; REM = Rapid Eye Movement Sleep. Percentages calculated by dividing the total number of minutes spent in each stage by the total sleep time.

To assess potential differences in sleep stages between the two experimental conditions, the total percentages of time spent in each of N1, N2, N3, and REM relative to TST were calculated (Table 1). The percentages of time spent in each of the different sleep stages were modelled as a function of experimental condition and sleep stage type. As displayed in Figure 2, our analysis revealed that there were no significant interactions between condition and the amount of time spent in each sleep stage, *b* = 0.0067, *t*(316) = −0.234, *p* = 0.82 (note that these results were confirmed by additional t-tests for all sleep stages, as well as arousals and sleep bout number and duration, all of which failed to detect significant differences in sleep characteristics between the two experimental conditions).

### 3.3. Ratings of Nap Quality and Sleepiness

**Figure 3:**
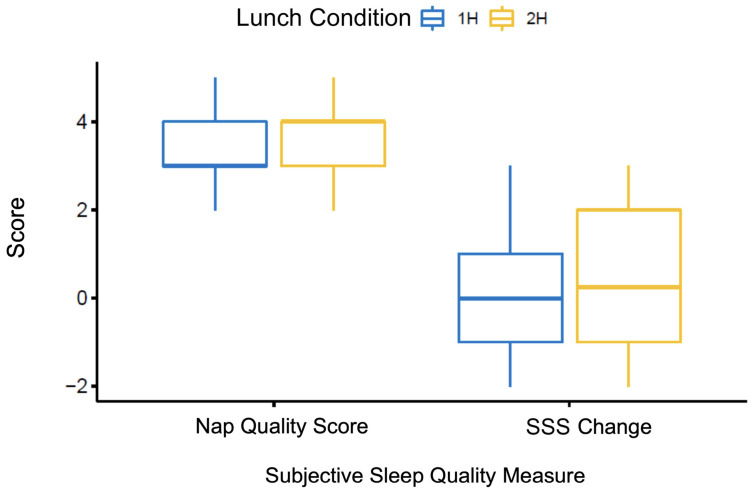
Subjective sleep quality measures by condition. Nap Quality Score = participants’ rating on the Subjective Sleep Quality Scale (SSQS; maximum of 5); Sleepiness Change = the participants’ pre-nap Stanford Sleepiness Scale (SSS) score subtracted by their post-nap SSS sleepiness score.

Two subjective questionnaire measures (Table 2) were completed by all participants: the Subjective Sleep Quality Questionnaire (SSQQ; ranging from 1 to 5, with higher scores indicating better nap quality) and the difference between pre- and post-nap SSS scores, calculated by subtracting the participants’ post-nap SSS score from their pre-nap SSS score. Thus, a larger, positive value indicated a larger reduction of sleepiness, whereas a larger, negative value indicated a greater increase of sleepiness. As displayed in Figure 3, our analysis revealed that neither the SSQQ scores nor the pre- and post-nap difference of SSS scores significantly differed between the 1 h and 2 h conditions, *t*(39) = −2.152, *p* = 0.04 and *t*(39) = −1.073, *p* = 0.290, respectively.

### 3.4. Analysis Check

As mentioned above, two participants were unable to fall asleep during their 1 h nap condition. To assess whether the results of the statistical analyses were influenced by these two participants, all analyses were repeated with data from these participants omitted from the data set (SOL—*t*(37) = −0.34413, *p* = 0.7327; TST—*t*(37) = −0.90322, *p* = 0.3723; SE—*t*(37) = −1.1144, *p* = 0.2723; Nap Quality—*t*(37) = −1.7809, *p* = 0.08315; SSS—t(37) = −1.2911, *p*-value = 0.2047; Sleep Stages as a function of Condition—*b* = −0.01321, *t*(300) = −0.529, *p* = 0.597). Given that none of the statistical results (i.e., lack of significant differences between experimental conditions) changed after removing these data, the participants who were unable to sleep during the nap condition were included in the data set and statistical analyses reported above.

## 4. Discussion

With the present set of experiments, we tested whether the time interval between eating lunch and an afternoon nap alters subjective and objective sleep measures. The results revealed similar sleep architecture, as well as similar subjective ratings of sleepiness and sleep/nap quality in participants completing the two experimental conditions (1 h and 2 h interval between lunch and a sleep opportunity). These findings contribute important, new information to the debate whether eating before sleep negatively impacts sleep by suggesting that, at least under the present experimental conditions, eating closer to the onset of an afternoon nap does not exert significant effects on several objective and subjective sleep parameters.

There is a large literature on the effects of food consumption in the evening or night on subjective and objective measures of overnight sleep, with most (but not all) studies reporting disturbances of sleep architecture and/or lower ratings of self-reported sleep quality following nightly eating [7,8,9,10,11,12,13,14,15,16,19,20,21,22]. For example, survey data have shown that eating within 3 h of nightly sleep onset is associated with increase in nocturnal awakenings [9], results that are in agreement with other survey findings of increased WASO with less time between an evening meal and nightly sleep onset [7]. Based on these and similar results, suggestions to avoid eating too close to bedtime are often included among sleep hygiene recommendations [17,18].

It is noteworthy that previous work so far has neglected to examine the question of potential effects of food intake on afternoon naps. Napping is widely recommended to counteract excessive daytime sleepiness, particularly in populations affected by poor nightly sleep quality (e.g., elderly, sleep disorder patients, but also shift workers, healthcare professionals [23,24,25,26,27,28]). To the best of our knowledge, the present study is the first to examine whether different time intervals between lunch and an afternoon nap alter sleep architecture and self-reports of sleep and sleepiness under controlled laboratory conditions.

Given the general assumptions and recommendations about food intake prior to sleep [17,18], it may be surprising that we did not detect significant differences in sleep architecture when the time interval from eating lunch to the start of the nap opportunity doubled from 1 h to 2 h. Afaghi et al. [10] employed polysomnography and found that increasing the delay between an evening meal and nightly bedtime from 1 to 4 h reduced SOL from about 14.6 to 9.9 min without altering any other aspects of sleep architecture. In contrast, we observed almost identical SOL between the two experimental conditions (14.5 and 14.3 min for the 1 h and 2 h conditions, respectively). Of course, the difference in the time interval conditions used, as well as the fact that Afaghi et al. [10] studied overnight sleep instead of a nap may account for the different effects on SOL between Afaghi et al. and the present set of experiments.

In a recent study, Duan et al. [11] used both conventional sleep staging and spectral power analysis to characterize changes in overnight sleep architecture in participants who consumed dinner either 1 or 5 h prior to bedtime. Similar to the results of the present investigation, sleep architecture assessed by conventional sleep staging was unaffected by the different time intervals [11]. Duan et al. noted a minor (2.5%), but significant increase in delta power early in the night, an effect which reversed during later sleep. The conclusions drawn by Duan et al. [11] that eating closer to sleep onset may not have adverse effect on overnight sleep architecture are similar to those that arise from the present study for afternoon napping: shortening the time interval between lunch and a nap from 2 to 1 h does not appear to exert obvious effects on sleep, as assessed by standard sleep staging measures, at least under the present experimental conditions.

In addition to polysomnography and sleep staging, we also asked participants to complete supplementary self-reports of sleepiness (the SSS) and the self-perceived quality of the nap. Given that various objective and subjective sleep measures do not always show high correlations among each other, we were particularly interested if one of the measures would exhibit a greater sensitivity to the effect of eating closer to sleep onset. However, neither the changes in sleepiness from pre- to post-nap SSS scores, as an indicator of the restorative effectiveness of the nap, nor the self-rated nap quality scores differed significantly between the two experimental conditions. Thus, overall, there was agreement among all experimental measures that the longer time interval between lunch and napping did not alter sleep characteristics.

### 4.1. Limitations and Future Direction

Our findings provide novel information to the literature on meal timing and sleep characteristics, and the experimental design has some clear strength. In particular, the repeated-measures design ensured that each participant acted as their own control, thus removing any systematic influence of stable participant characteristics on the outcome variables (e.g., chronotype, typical sleep–wake schedule and social jet lag, various personality traits, age, as well as eating- and metabolism-related factors). Similarly, any potential effect of nutrient content (such as glucose content, which has been linked to sleep quality and insomnia [29,30]) on sleep quality was controlled for, given that participants consumed identical meals during both experimental sessions.

At the same time, it is also important to consider the limitations of this study. Data collection occurred between August 2021 and February 2022, i.e., during the COVID-19 pandemic. As such, this study faced challenges in terms of participant recruitment due to local and institutional health guidelines. In addition, the main demographic of our sample was young adults, most of whom were enrolled as students at Queen’s University. Therefore, the findings of this study likely are limited in their generalizability to a wider and more diverse population. For instance, it is well documented that sleep architecture and circadian rhythms change across the lifespan [31]. Due to these age-related changes, it is possible that our results would not replicate in a sample of middle-aged or elderly populations. Further, while our sample size (n = 40) is larger than similar work using in-lab polysomnographic techniques (e.g., n = 20 in [11]), a larger sample size may facilitate the detection of smaller differences in sleep characteristics following meal consumption. It is also noteworthy that the majority of the participant sample was female (n = 29, 73%); this unbalanced representation of males and females, and the resulting small sample size for males (n = 11) precluded any formal statistical assessment of potential sex differences (even though informal data inspection did not suggest any obvious differences in our data set). To address some of these concerns, future studies should aim to replicate this study with a larger, more diverse sample (e.g., age, ethnic background, gender). In addition, since it is possible for numerous factors to interact with food intake to alter sleep characteristics, follow-up work should also collect more detailed information regarding participant characteristics, including, but not limited to, menstrual cycles in females, sociodemographic information, as well as levels of stress and psychological well-being.

As a further limitation, we did not monitor or attempt to control food intake prior to the experimental sessions in the lab, given that our objective was to assess the specific role of the time interval between lunch and napping on sleep, rather than of food intake over a longer time period (e.g., the entire day). We instructed participants to consume as much of the lunch as possible, without reaching the point of discomfort. Since we did not otherwise instruct participants to alter their meal schedules, it is likely they arrived at the lab with varying levels of hunger and caloric needs. As such, we expected participants to consume as much of the meal as they could in order to reach their optimal level of satiety. While we purposefully did not attempt to study the influence of these variables, it is nevertheless possible that they exert effects on nap characteristics, and future work should consider the combined influence of meal timing, longer-term food intake, and satiety levels on the quality of both nightly sleep and afternoon naps.

We administered the SSS at two time points, shortly before the nap opportunity and after the nap, following the removal of all electrodes and cleaning of the participants scalp and hair. The SSS was used in order to obtain a measure of subjective sleepiness in our sample prior to the nap, as well as to track potential changes (e.g., decreases) in sleepiness from the time before the nap to the time after the nap was completed. We hypothesized that better sleep quality would lead to a greater difference score between pre- and post-nap SSS scores, indicative of a more effective relief of daytime sleepiness.

As mentioned, our results did not reveal significant differences in SSS between the experimental conditions and also did not show any clear change between the two time points of SSS administration. A concern with SSS measures following periods of sleep is the potential influence of sleep inertia, i.e., a feeling of continued tiredness that is associated with impaired cognitive performance [32]. Given the length of our nap opportunity (2 h, with TST of about 54 and 59 min in the 1 h and 2 h lunch conditions, respectively), it is likely that some level of sleep inertia was present after the nap, which may have influenced post-nap SSS rating. We attempted to reduce sleep inertia by engaging with the participant during the post-nap clean up (about 10–15 min) before administering the post-nap SSS questionnaire. However, the potential that lingering sleep inertia affected SSS rating is a limitation of the present experiments. Future studies could use shorter nap opportunities, or longer time intervals between the end of the nap and SSS administration to reduce sleep inertia and, thus, overcome these problems.

We also purposefully decided not to provide instructions to the participants to adhere to a specific sleep schedule on the nights prior to the experimental sessions. Instead, we encouraged everyone to continue with their regular schedule of sleeping and eating to ensure that any results of this investigation were valid in the context of the typical lifestyle of this specific demographic (mostly undergraduate university students). Of course, the absence of a regulated sleep–wake cycle introduces confounds, such as the potential for high levels of tiredness during the experimental sessions. Mean baseline (e.g., pre-napping) scores of SSS were 3.2 in our sample, which are similar to those reported in the literature for similar demographic populations [33,34,35], thus suggesting that, on average, our participants did not suffer from excessive sleepiness. Nevertheless, future extensions of the present work may consider imposing a regular sleep schedule on the nights leading up to the experiment to better control the potential impact of differential sleepiness levels on the experimental measures.

Even though our results suggest that sleep architecture and subjective rating of sleep and sleepiness do not change as a result of eating lunch 1 h vs. 2 h prior to a nap, more research is clearly needed in order to confirm and expand upon the present findings. For example, time intervals shorter and longer than those used here should be examined to further characterize the specific role of timing of food intake and afternoon sleep. In addition, future studies could manipulate additional factors that have been suggested to affect sleep quality, such as caloric and nutrient content [10,19,20,21]. It is also conceivable that the timing and content of food can interact to impact sleep, necessitating studies that manipulate all of these variables (even though such work will be relatively complex in terms of the experimental design). Finally, as discussed above, some prior work has detected minor, but significant changes in specific frequency bands in overnight EEG activity following an evening meal [11]. Thus, future work could extend the analyses of EEG data obtained during napping by including additional measures such as spectral power analyses to gain a better understanding of changes in restricted EEG frequency bands following lunch consumption.

### 4.2. Conclusions

In summary, our investigation on the impact of eating lunch 1 or 2 hours prior to the onset of an afternoon nap revealed similar levels of subjective and objective sleep quality between the two conditions. These findings are important, as afternoon napping is associated with a number of beneficial effects, including improvements in cognitive functioning, attention, learning and memory performance; reduced fatigue; and increased mood [26,27,28]. Consequently, obtaining regular naps is often recommended, specifically for individuals who suffer from chronic sleep debts due to poor or insufficient overnight sleep and/or irregular sleep–wake schedules, such as the elderly, sleep disorder patients, healthcare professionals, or shift workers [23,24,25,26,27,28]. Notably, adolescents and young adults (including university/college students who made up the majority of our sample) are also known to suffer from insufficient and poor-quality sleep, as well as significant social jet lag [36,37,38]. Some experts consider adolescents/young adults as a “vulnerable population” for poor sleep health [2], making them an additional target population who could benefit from incorporating naps into their daily routine. As such, understanding if and how the time interval between lunch consumption (which often occurs within a few hours of the typical napping period) and sleep onset influences nap quality is important, as it can inform these target populations on best practices to obtain high-quality afternoon naps. Our research suggests that, at least within the parameters of the present study, the precise timing of napping in relation to eating lunch may not exert a major influence on nap quality. Consequently, and if confirmed by future work, these findings imply that individuals would not have to be overly concerned about adhering to a highly specific schedule for lunching and napping, and that restorative naps are possible even when a meal has been consumed a relatively short time interval (e.g., 1 h) prior to a nap opportunity. Future research should continue to identify factors that impact nap quality, with the aim of formulating a validated set of recommendations for optimal nap hygiene that can be used in both professional (e.g., workplace, schools) and home environments.

## 5. Methods

### 5.1. General Experimental Design

**Figure 4:**
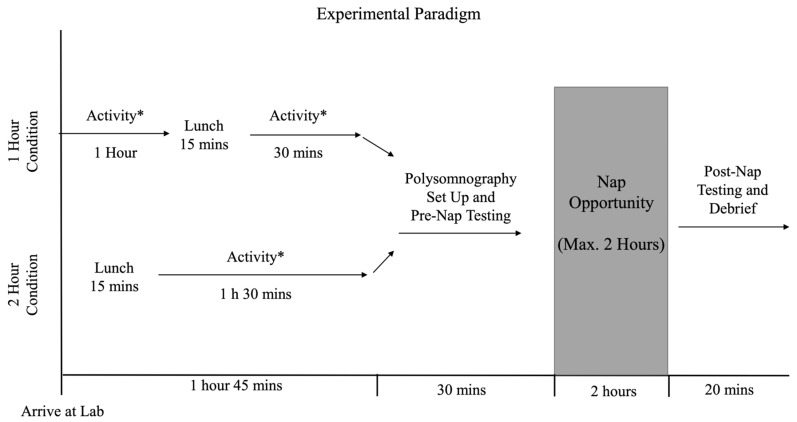
Timeline of experimental procedures. * All activities performed were sedentary (e.g., reading, homework) and each participant performed the same activities for both the 1 h and 2 h condition (please note that the grey area indicates the time window for the nap opportunity).

The study employed a randomized within-subject (repeated-measures) design where each participant completed both experimental conditions (1 h and 2 h interval between lunch and the nap opportunity; in randomized order; 7 days apart). Prior to the two experimental sessions, all participants also came to the laboratory for an initial visit to receive information about the study and provide informed, written consent. The study design, experimental conditions, and timeline of all experimental procedures are summarized in Figure 4.

This study was reviewed and approved by the General Research Ethics Board (GREB) at Queen’s University in Kingston, ON, Canada. All participants gave informed, written consent prior to the commencement of data collection.

### 5.2. Participants

A total of 40 participants were recruited and completed the study after providing informed, written consent prior to data collection (see Results Section for a detailed breakdown of participant characteristics). Participants were recruited from the Kingston, Ontario, Canada area by means of social media posts (Facebook), posters displayed on-campus and in the Kingston community, as well as through the official Psychology Department Participant Pool (which provides course credits for students enrolled in undergraduate Psychology courses). Participants were not considered for the study if they met any of the following criteria: current diagnosis of a sleep or psychiatric disorder; diagnosis of diabetes and/or a circadian rhythm or metabolism disorder. Further, participants who had severe food allergies or were unable to give informed consent were also not eligible for the study. Additionally, participants were required to engage in regular napping, defined as an average of at least one nap per week for the last two months.

### 5.3. Experimental Protocol

All participants completed a total of three visits to the laboratory: Visit 1: Study information and consent; Visit 2: Experimental session #1 (1 h or 2 h time interval between lunch and the sleep opportunity, randomly assigned for each participant); Visit 3: Experimental session #2 (the second time interval for each participant). The period between the two experimental sessions was seven days, which ensured that participants came on the same weekday and likely had similar schedules (school, work, extracurricular activities, etc.) on both experimental days, as well as the day and night preceding the experiment. However, two participants had visits which occurred several weeks apart, due to the outbreak of the omicron variant of COVID-19 that occurred in Kingston, Ontario from late December 2021–mid January 2022, which resulted in the suspension of all in-person research activities.

#### 5.3.1. Visit 1: Study Information and Consent

Interested participants were invited to the lab for a 30 min visit in order to provide them with detailed information about the study and allow them to become familiar with the laboratory and sleep environment. During this visit, participants provided written, informed consent and filled out a brief demographic questionnaire (e.g., age, gender, general sleep quality, dietary restrictions). As part of the questionnaire, participants (who all were regular nappers; see above) were asked to indicate the time when they would typically have a nap in their regular environment. The two subsequent experimental sessions were scheduled so that each participant would be able to have their nap at around their typical nap time (e.g., if a participant typically napped around 3 pm, the experimental sessions would start at about 12:45 p.m., that is, 2:15 h prior to the start of the nap opportunity in the lab, in accordance with the timeline of the experimental protocol; Figure 4). The two experimental sessions were scheduled on workdays at identical times for each participant, and the typical time interval between these sessions was 7 days.

In addition, participants selected their lunch choices for the two subsequent experimental sessions (1 h and 2 h time interval between lunch consumption and the onset of the nap opportunity; see below for details). Next, participants were then given the opportunity to enter the room where they would be sleeping in order to familiarize themselves with the setting and reduce potential discomfort about sleeping in an unfamiliar place during the actual testing days. In addition, participants were informed that they could bring any sleep-related items (e.g., pyjamas, pillows, blankets) from home so that they would feel more comfortable during the actual napping opportunities. All participants were screened for COVID-19 symptoms prior to being permitted entry to the lab and were required to wear a mask during all visits to the lab (except during the actual nap), as per public health guidelines. Participants were not instructed to adhere to any specific sleep schedule prior to their subsequent experimental sessions; rather, we asked participants to continue their usual daily and nightly routines in terms of eating, sleeping, and napping.

#### 5.3.2. Visit 2: Experimental Session #1

For both experimental sessions, participants spent about 2 h 15 min in the laboratory prior to the start of the nap opportunity (Figure 4). During this time, participants were asked to engage in a sedentary activity that they would normally perform during this time of their day (i.e., doing homework, reading, watching a movie; same activity for both experimental sessions).

Lunch Conditions: In addition, during this time prior to the nap opportunity, participants were served lunch (about 15 min to consume the meal) so that they finished eating either 1 or 2 h prior to the onset of the nap opportunity (Figure 4). Lunches included a sandwich, one side dish, and one drink. Sandwiches were obtained from a commercial chain (Subway, franchise location in Kingston, ON, Canada) and consisted of a 6-inch sandwich on either Italian- or multigrain bread. Participants could choose from the following sandwich options: turkey (280 calories, 43 g carbohydrates, 5 g fat, 19 g protein); ham (280 calories, 43 g carbohydrates, 5 g fat, 18 g protein); cold cut (400 calories, 43 g carbohydrates, 18 g fat, 17 g protein); or vegetable (210 calories, 39 g carbohydrates, 3 g fat, 10 g protein; obtained from www.subway.com/en-ca/menunutrition/nutrition; last accessed on 4 August 2024). Participants were free to add any additional vegetable toppings and sauces (not included in the nutrient information provided above). Side dishes were obtained from a local grocery store and included: vegetables and dip (approx. 200 g), an apple (approx. 100 g), a granola bar (26 g), or a fruit cup (107 mL). Drink options (obtained at Subway or local grocery store) were: 1% white milk (250 mL), orange juice (200 mL), apple juice (200 mL), or water (500 mL). In order to allow participants more choice and respect dietary preferences and restrictions, lunches were not matched for precise calories or nutrient content. However, and importantly, each participant received identical lunches for their two experimental sessions, thus allowing each participant to act as their own control and removing any systematic effect of the specific meal content on the outcome variables.

At about 30 min prior to the nap opportunity, all participants were given the opportunity to change into more comfortable clothes for their nap, followed by being prepared for the polysomnography recordings.

Polysomnography: Electroencephalographic (EEG) activity was recorded using a bipolar montage, with EEG electrodes (Genuine Grass Gold Disc electrodes; Natus Medical, Middleton, WI, USA) placed on the scalp in accordance with the International 10–20 System at Fz-Cz and Cz-Oz. To measure eye movement (electrooculogram, EOG), two electrodes were placed near and lateral to the two eyes (outer canthus), with one electrode (E1) positioned approximately 1 cm below and lateral to the left eye, and a second electrode (E2) positioned about 1 cm above and lateral to the right eye. Finally, two electrodes were placed on the masseter to measure muscle activity (electromyogram, EMG) of the jaw and chin, and three additional electrodes were placed immediately behind the ears over the mastoid process to serve as ground and reference (for E1 and E2) connections. Physiological activity was amplified (0.3 Hz and 10 kHz), digitized (200 Hz; PowerLab/30 system running LabChart software, v. 8. 1. 11, ADInstruments, Toronto, ON, Canada), and stored for subsequent analyses (see below).

Stanford Sleepiness Scale (SSS): Following the polysomnography preparation, participants completed the SSS [39]. The SSS is a single-item scale that assesses current levels of sleepiness along a 7-point scale, ranging from “Feeling active, vital, alert, or wide awake” (1 point) to “No longer fighting sleep, sleep onset soon; having dream-like thoughts” (7 points). The SSS was administered twice: after the PSG preparation prior to the nap (pre-nap), and about 15 min following the nap and the PSG clean-up (post-nap).

Nap Opportunity: Next, participants were brought to the private bedroom, where they were asked to remove their mask, lie down, and go to sleep. The experimenter left the room and turned off the lights, which signaled the start of the polysomnographic recordings. The nap opportunity lasted for a maximum of 2 h and participants who were still asleep after the 2 h had elapsed were awoken by the experimenter.

Post-Nap Procedures: Following the nap opportunity, participants were instructed to put on their face mask and were brought back into the main laboratory to remove all electrodes and clean their skin and hair of residual adhesive and conductive paste. The cleaning procedure (about 10–15 min) was also used to actively engage with the participant in order to counteract and dissipate any possible sleep inertia that may have been present after the nap. Subsequently, participants were asked again to complete the SSS, as well as a Subjective Sleep Quality Questionnaire (SSQQ), an in-house-developed 5-item tool specifically designed to allow a direct comparison between objective sleep architecture measures and subjective sleep assessments. As such, the SSQQ includes the following items: whether they think they slept or not; an estimate for how long they slept; estimates of SOL; the number of times they woke up during the nap (all of which can be directly compared to objective sleep architecture measures). An additional item asks for a rating of sleep quality along a 5-point Likert scale, with 1 and 5 indicating the poorest and excellent sleep/nap quality, respectively. For the purpose of the present study, only the item assessing nap quality was included in the data analyses.

After the completion of the questionnaires, all participants were debriefed and compensated for their time.

#### 5.3.3. Visit 3: Experimental Session #2

All procedures for Experimental session #2 were identical to those of session #1, with the exception that the time interval between eating lunch and the start of the nap opportunity was now reversed; participants who were in the 1 h condition now consumed their meal 2 h before nap time and vice versa (see Figure 4).

### 5.4. Polysomnographic Scoring

All polysomnographic recordings were scored offline in accordance with the American Academy of Sleep Medicine guidelines [40]. Each recording was scored by two independent, trained scorers who were blind to the experimental condition of the recording; in cases of disagreement between the two scorers, a third (blind to condition) scorer was consulted. All recordings were analyzed to identify sleep onset latency (SOL), total sleep time (TST), sleep efficiency (SE), number of arousals, and the total time and percentage of the overall sleep period that was spent in each sleep stage (N1, N2, N3/SWS, and REM). For the identification of delta/slow wave activity and N3 scoring, we also paid close attention to the frequency component (e.g., dominant 1–4 Hz activity), in addition to the amplitude criterion (≥75 microvolt) typically recommended for N3 scoring [40].

### 5.5. Statistical Analyses

All statistical analyses were conducted using version 4.1.3 of R [41]. For the purposes of these analyses, 9 variables were examined between the experimental conditions (1 h vs. 2 h interval): TST, SOL, SE, the difference between pre-nap SSS scores and post-nap SSS scores, subjective nap quality ratings, and the percentage of the sleep spent in each of N1, N2, N3, and REM. In total, 3 multilevel models were conducted.

Prior to analysis, data winsorization was performed separately on each of our three datasets (objective sleep quality; sleep architecture; and subjective sleep quality). Within each dataset, the following number of outliers were identified: 4 in the objective data (2 within the SOL, 2 within the SE); 4 in the subjective data (all 4 in for nap quality rating using the SSQQ); and 19 within the sleep architecture data (2 for N1%; 2 for N2%; 5 for N3%, and 10 for REM%). The 19 outliers in the sleep architecture data could be attributed to large variance in the amount of time experienced within each sleep stage. This is especially true considering that not every napper entered each sleep stage. To eliminate these outliers, 90% winsorization was used: 90% winsorization replaced all scores above the 95th percentile, and below the 5th percentile, with the next closest score. Winsorization can act to reduce the levels of an experimental variable (e.g., by replacing the most extreme levels at either end of an experimental measure or scale), which may be problematic for variables with a relatively low number of levels (e.g., five levels for the SSQQ). However, here, only 4 data points for the SSS were replaced. In addition, given that a larger number of outliers were identified for the sleep architecture variables, we also conducted an additional analysis check for these measures to ensure that the winsorization did not impact the results. Our results revealed no significant change in statistical results when using winsorized variables or non-winsorized variables. 

For the formal statistical analyses of objective sleep quality measures (TST, SOL, and SE), we ran three paired t-tests to determine if these measures differed between the two experimental conditions, using the Bonferroni correction method (new *p* = 0.01). (Prior to running paired t-tests, the data for the sleep architecture measures were modelled as a function of experimental condition and sleep architecture measure type using a 2-level, random intercept multilevel model. All interactions between condition and objective sleep measures were non-significant >0.05).

Similarly, all sleep stage data were winsorized (to reduce the impact of outliers) prior to analysis and experimental condition was contrast coded so that the 1 h condition was coded as ‘0.5’ and the 2 h condition was coded as ‘−0.5’. Sleep stage type was coded as follows: N1 was coded as ‘0’, N2 was coded as ‘1’, N3 was coded as ‘2’, and REM was coded as ‘3’.

To determine how the percentage of time spent in each sleep stage varied between the two experimental conditions, we ran a 2-level, random intercept multilevel model using the ‘lmer’ function from the *lme4* package [42] in R [41], which by default used an unstructured covariance matrix and Satterthwaite’s method for determining degrees of freedom. For sleep stage data, we only reported the multilevel model results (and not t-test results), given that multilevel modeling is a more appropriate and conservative analysis for proportional data (i.e., as one sleep stage increases, another will often decrease), controls for multiple comparisons and is more robust against violations of the assumptions of parametric statistics, which were present in this data set. We did, however, conduct additional t-tests on sleep stage data, all of which confirmed the multilevel model results.

As mentioned above, prior to analysis of questionnaire data (SSS and SSQQ), data for each of the questionnaire variables were winsorized to reduce the impact of outliers. To determine how questionnaire scores varied between the two experimental conditions, we ran two paired *t*-tests using the Bonferroni correction (adjusted *p* = 0.01). (Prior to running the t-tests, the scores for the two questionnaires were modelled as a function of experimental condition and questionnaire measures using a 2-level, random intercept multilevel model. All interactions between condition and questionnaire scores were non-significant, *p* > 0.05).

## Figures and Tables

**Table 1:** Sleep architecture.

Measure	1 h Condition M (SD)	2 h Condition M (SD)	Cohen’s *d*
SE (%)	60.0 (25.7)	65.3 (25.3)	0.22
TST (mins)	53.5 (30.7)	58.9 (30.7)	0.18
SOL (mins)	14.5 (11.9)	14.3 (11.0)	0.01
N1 (% of TST)	18.6 (15.6)	18.5 (18.0)	0.002
N2 (% of TST)	58.1 (23.9)	59.1 (22.9)	0.04
N3 (% of TST)	13.0 (19.5)	17.3 (20.4)	0.2
REM (% of TST)	5.1 (8.3)	5.2 (10.2)	0.002

SE: sleep efficiency, the total amount of time participants slept divided by the total amount of time participants spent in bed. TST: total sleep time, the total amount of time participants slept. SOL: sleep onset latency, the amount of time it took participants to fall asleep. M: mean. SD: standard deviation.

**Table 2:** Questionnaire measures.

Sleep Measure	1 h Condition M (SD)	2 h Condition M (SD)	Cohen’s *d*
SSQQ	3.3 (1.1)	3.7 (0.9)	0.34
Pre-SSS—Post SSS	0.1 (1.6)	0.4 (1.6)	0.17

Nap Quality: participants’ scores on the Subjective Sleep Quality Questionnaire (SSQQ); Pre-SSS—Post-SSS: the difference between participants’ pre- and post-nap ratings on the Stanford Sleepiness Scale (SSS); M: mean; SD: standard deviation.

## Data Availability

The data for the study presented in this article will be shared on reasonable request to the corresponding author.
